# Autophagic degradation of CDK4 is responsible for G0/G1 cell cycle arrest in NVP-BEZ235-treated neuroblastoma

**DOI:** 10.1080/15384047.2024.2385517

**Published:** 2024-08-01

**Authors:** Zhen Liu, Xiao-Yang Wang, Han-Wei Wang, Shan-Ling Liu, Chao Zhang, Feng Liu, Ying Guo, Feng-Hou Gao

**Affiliations:** a Department of Clinical Laboratory, Shanghai First Maternity and Infant Hospital, School of Medicine, Tongji University, Shanghai, China; b Department of Oncology, Shanghai ninth People’s Hospital, Shanghai Jiao Tong University School of Medicine, Shanghai, China; c Department of Blood Transfusion, The Sanmenxia Central Hospital, Sanmenxia, Henan Province, China; d Department of Rheumatology and Immunology, Bengbu Third People’s Hospital Affiliated to Bengbu Medical College, Bengbu, Anhui, China; e Department of Clinical Laboratory, The First Hospital of Changsha City,Changsha, Hunan, China; f Department of Geriatrics, Shanghai ninth People’s Hospital, Shanghai Jiao Tong University School of Medicine, Shanghai, China; g Department of Pathology, Yellow River Hospital Attached Henan University of Science and Technology, Sanmenxia, Henan Province, China

**Keywords:** CDK4, autophagic degradation, neuroblastoma, P62, NVP-BEZ235

## Abstract

**Background:**

CDK4 is highly expressed and associated with poor prognosis and decreased survival in advanced neuroblastoma (NB). Targeting CDK4 degradation presents a potentially promising therapeutic strategy compared to conventional CDK4 inhibitors. However, the autophagic degradation of the CDK4 protein and its anti-proliferation effect in NB cells has not been mentioned.

**Results:**

We identified autophagy as a new pathway for the degradation of CDK4. Firstly, autophagic degradation of CDK4 is critical for NVP-BEZ235-induced G0/G1 arrest, as demonstrated by the overexpression of CDK4, autophagy inhibition, and blockade of autophagy-related genes. Secondly, we present the first evidence that p62 binds to CDK4 and then enters the autophagy-lysosome to degrade CDK4 in a CTSB-dependent manner in NVP-BEZ235 treated NB cells. Similar results regarding the interaction between p62 and CDK4 were observed in the NVP-BEZ235 treated NB xenograft mouse model.

**Conclusions:**

Autophagic degradation of CDK4 plays a pivotal role in G0/G1 cell cycle arrest in NB cells treated with NVP-BEZ235.

## Introduction

Neuroblastoma (NB) is the most common pediatric solid tumor arising from neural crest precursors, and it is a heterogeneous disease ranging from spontaneous regression to high risk of fatality.^1^ The less than half of children with high-risk NB survive despite intensive multimodal therapy.^2^ It is believed that NB have defects in regulation of cellular differentiation, in particular G1 progress and G1/S transition.^3,4^ Recent studies have addressed that CDK4 is highly expressed or hyperactivated, and it has been identified as a key driver of proliferation in various human cancers, including NB.^5–7^ D’Oto et al. reported the amplification of CDK4 was found in a high-risk NB subgroup (MYCN-amplified NB), which suggested its upregulation is correlated with the tumorigenesis, progression and poor prognosis of NB.^8^ Hence, the strategy targeting CDK4 may become a promising therapeutic approach in NB.

The highly selective CDK4 inhibitors, such as Palbociclib and Ribociclib, have already shown significant antitumor activity against NB in several pre-clinical or clinical trials.^9–11^ These compounds can lead to competitive inhibition of active CDK4 via interacting with the ATP-binding pocket of CDK4. However, the existence of distinct non-CDK4 targets have been reported to explain the therapeutic failures including acquired resistance and hematological toxicity,^12,13^ which are major obstacles for widespread implement. The compounds that degrade CDK4 rather than inhibit its enzymatic activity are indispensable and innovative, especially in overexpressed and continuously activated NB. The ubiquitin proteasomal-dependent CDK4 degradation is widely known for the inducible proliferation inhibition.^14,15^ However, it has not been mentioned about autophagic degradation of CDK4 protein and its anti-proliferation effect in NB cells.

SH-SY5Y and SK-N-MC cells have been widely used in neuroblastoma research^16,17^and our previous study has shown NVP-BEZ235 (BEZ235), a dual PI3K and mTOR inhibitor^18^ declined the expression of PI3K/mTOR pathway-related proteins in both cells.^19^ Therefore, both cells were used to evaluate the roles and mechanism of autophagic degradation of the CDK4 on cell cycle distribution. Our present results indicated that BEZ235 facilitates autophagic degradation of CDK4, which can rapidly induce NB cell cycle arrest at G0/G1 state and proliferation inhibition. Significantly, P62/cathepsin B (CTSB) are required for autophagic degradation of CDK4 induced by BEZ235 against NB in vitro and in vivo. These results not only establish the pivotal role of the autophagy pathway in CDK4 turnover but also suggest the potential application of BEZ235 or other drugs via the therapeutic modulation of autophagic degradation of CDK4 protein in NB.

## Results

### BEZ235 induced G0/G1 cell cycle arrest with the downregulation of CDK4 protein in NB cells

**Figure 1.**
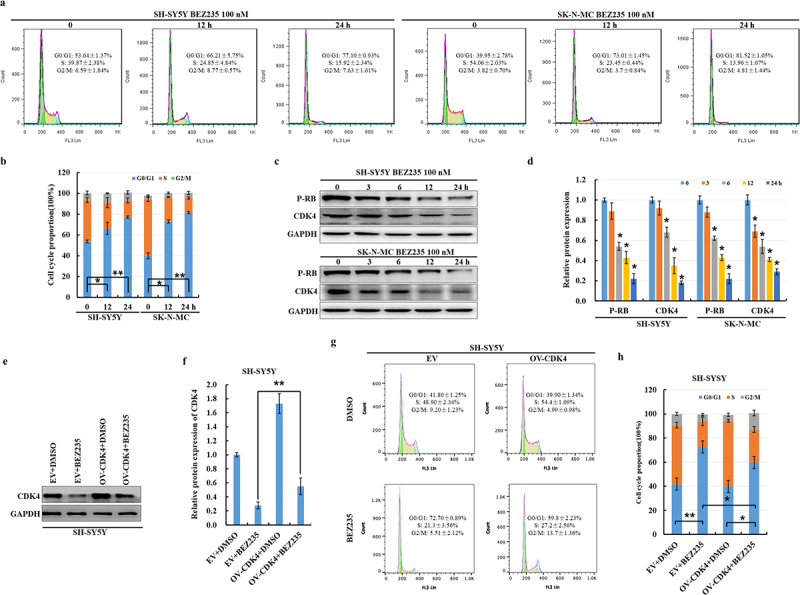
BEZ235 induced G0/G1 cell cycle arrest with the downregulation of CDK4 protein in NB cells. (a-b) SH-SY5Y and SK-N-MC cells were treated with 100 nM BEZ235 for 0, 12 h, and 24 h. The cell cycle distribution was assessed by flow cytometry analysis. (c-d) SH-SY5Y and SK-N-MC cells were treated with 100 nM BEZ235 for the indicated time. The CDK4 and p-RB proteins were analyzed by immunoblotting and the results were normalized to GAPDH by quantitative the optical density of the protein bands. (e-h) SH-SY5Y cells were transfected with pcDNA3.1 empty (EV) and overexpressed (OV)-CDK4 for 48 h and then treated with DMSO or 100 nM BEZ235 for another 12 h, the CDK4 levels were analyzed by immunoblotting, the results were normalized to control group (EV+DMSO), and cell cycle distribution was detected by flow cytometric analysis.

We analyzed the effect of BEZ235 on cell cycle distribution in NB cells via flow cytometry using PI staining of the DNA content. After exposure to 100 nM BEZ235 for 0, 12, and 24 h, there was a remarkable increase in the percentage of the NB cells in the G0/G1 phase compared with the control group (Figure 1a,b). Then, we assessed the expression of endogenous CDK4 and downstream phospho-retinoblastoma (p-Rb) proteins when SH-SY5Y and SK-N-MC cells were treated with 100 nM BEZ235 for various times. As shown in Figure 1c and d, there was a gradual decrease in CDK4 and p-Rb protein levels in a time-dependent manner. Additionally, the overexpression of CDK4 can reverse the degradation of CDK4 and decrease the percentage of BEZ235-treated cells in the G0/G1 phase (Figure 1e–h). These data suggested that BEZ235 induces notable G0/G1 cell cycle arrest in NB cells through the downregulation of CDK4 protein.

### BEZ235 attenuated CDK4 protein levels by facilitating protein degradation

**Figure 2.**
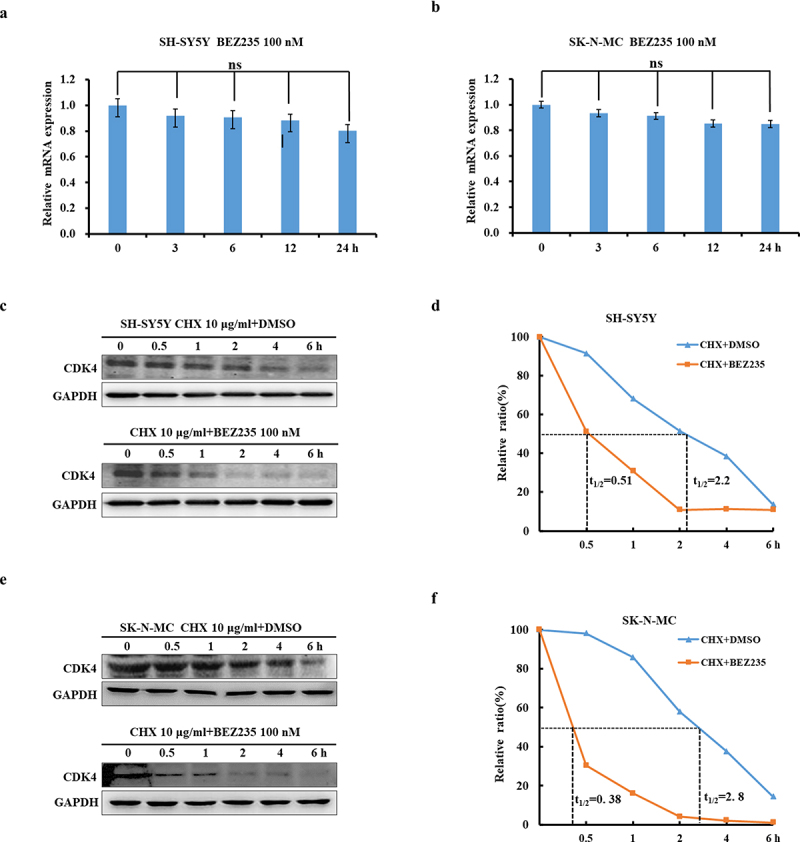
BEZ235 decreased CDK4 levels by enhancing its protein degradation. (a, b) SH-SY5Y and SK-N-MC cells were incubated with BEZ235 for the indicated time. CDK4 mRNA levels were determined by RT-PCR. (c-f) SH-SY5Y and SK-N-MC cells were pretreated with DMSO or 100 nM BEZ235 for 1 h, and then 10 μg/ml CHX was added for various times. The CDK4 protein levels were detected by immunoblotting and quantified by densitometry. The results were normalized to GAPDH.

To further illustrate whether BEZ235-mediated downregulation of CDK4 protein was associated with transcriptional or posttranscriptional regulation, the CDK4 mRNA levels were evaluated by RT-PCR. As indicated in Figure 2a and b, the mRNA levels of CDK4 were not drastically altered in the presence of 100 nM BEZ235. This indicates that BEZ235 may promote the protein degradation of CDK4 that was evaluated by CHX-chase analysis. As shown in Figure 2c–f, NB cells were cotreated with 10 μg/ml CHX for various times followed by pretreatment with 100 nM BEZ235 for 1 h. The half-life of CDK4 protein were obviously reduced in both BEZ235 treated cell lines. Based on the above results, we concluded that BEZ235 attenuates CDK4 protein stability and promotes its degradation.

### Autophagy-lysosome inhibitors can partially reverse the degradation of CDK4 protein and G0/G1 cell cycle arrest induced by BEZ235

**Figure 3.**
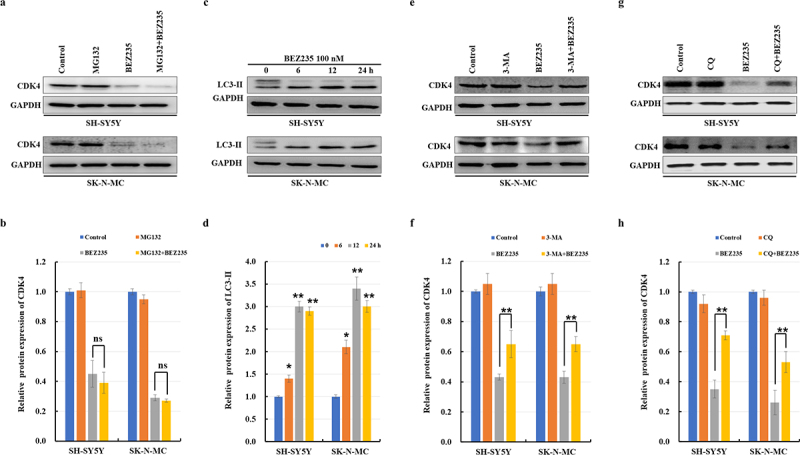
The autophagy-lysosome pathway was involved in BEZ235-induced degradation of the CDK4 protein. (a,b) SH-SY5Y and SK-N-MC cells were pretreated for 1 h with 10 μM proteasome inhibitor (MG132) and subsequently treated for another 12 h with 100 nM BEZ235, then the lysates were probed for CDK4 antibodies by immunoblotting. (c,d) Western blot analysis of the LC3 protein in SH-SY5Y and SK-N-MC cells treated with 100 nM BEZ235 for various times. (e-h) NB cells were pretreated for 1 h with an autophagy inhibitor (20 μM CQ or 2 mM 3 MA) and subsequently treated for another 12 h with 100 nM BEZ235. Western blot analysis was performed to analyze CDK4 proteins. The results were normalized to control group.

To clarify which pathway is involved in the degradation of CDK4 protein, ubiquitin-proteasome and autophagic degradation pathways were assessed via the respective inhibitors. First, we pretreated NB cells with the 26S proteasome inhibitor MG132 (10 μM) for 1 h, followed by treatment with 100 nM BEZ235 for another 12 h. We observed the CDK4 protein did not change significantly when MG132 in combination with BEZ235 compared with the single BEZ235 group (Figure 3a,b). Next, we further assessed autophagy-related hallmark LC3 protein in both cell lines treated with BEZ235. As shown in Figure 3c,d BEZ235 enhanced the conversion of LC3-II at an early stage, which suggests autophagic pathway might play a role in the CDK4 protein degradation. Then, the effects of the autophagy inhibitors CQ and 3-MA were analyzed. Intriguingly, cotreatment with CQ or 3-MA ameliorated the expression of CDK4 and G0/G1 cell cycle arrest induced by BEZ235 (Figures 3e–h and 1).

### The knockdown of autophagy-related genes could partially reverse the CDK4 protein degradation and G0/G1 cell cycle arrest mediated by BEZ235

**Figure 4.**
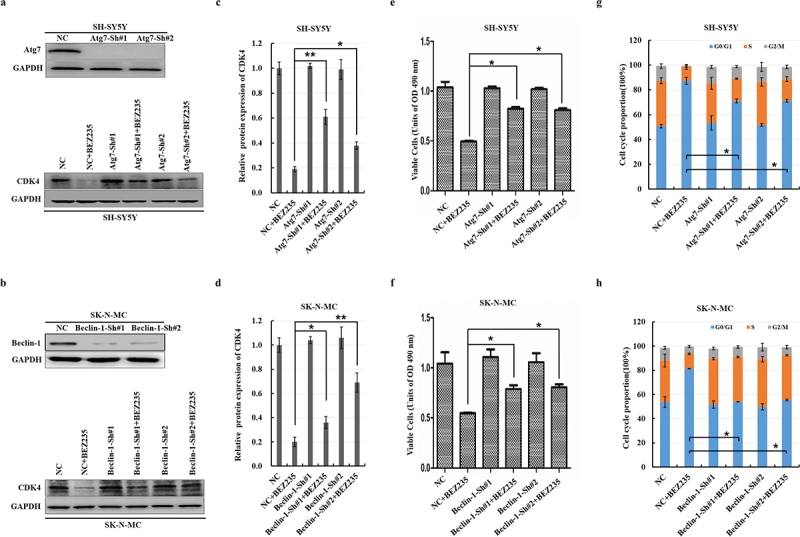
The autophagic degradation pathway was indispensable for BEZ235-induced CDK4 degradation. (a,b) SH-SY5Y and SK-N-MC cells were transfected with shRNA specifically targeting *Atg7* or *beclin-1* and then assessed by immunoblotting with the respective antibodies. Transfected cells with individual shRNAs were subsequently treated for 12 h with DMSO or 100 nM BEZ235. The indicated CDK4 proteins were analyzed by immunoblotting. (c,d) the results were normalized to NC group. (e,f) cell viability was assessed using the CCK-8 assay. (g,h) cell cycle distribution was determined by flow cytometric analysis.

Autophagy is one of the two major intracellular protein degradation routes within eukaryotic cells. To further verify whether BEZ235-induced autophagy influences NB cells, we tested the effect of the autophagic inhibition process on CDK4 degradation, cell viability and cell cycle distribution via knockdown of autophagy-related genes. After transfection with shRNA targeting *Beclin-1* or *Atg7*, NB cells were treated with DMSO or 100 nM BEZ235 for 12 h. CDK4 was analyzed by immunoblotting, cell viability was assessed using a CCK-8 assay, and cell cycle distribution was demonstrated with flow cytometry analysis. We found that blockade of autophagy markedly reversed the CDK4 protein levels (Figure 4a–d). Simultaneously, the viability of BEZ235-treated NB cells was obviously improved (Figure 4e,f), and the percentage of BEZ235-treated cells in the G0/G1 phase was partially decreased (Figure 4g and Fig. s2a) in SH-SY5Y cells. Surprisingly, there is the fully restored cell cycle profile in SK-N-MC cells (Figure 4h and Fig. s2b) due to autophagy inhibition. These results demonstrated that the autophagy-mediated CDK4 degradation plays roles in BEZ235-induced G0/G1 cell cycle arrest and proliferation inhibition in NB cells.

### P62 and cathepsin B are indispensable for autophagic degradation of the CDK4 protein induced by BEZ235

**Figure 5.**
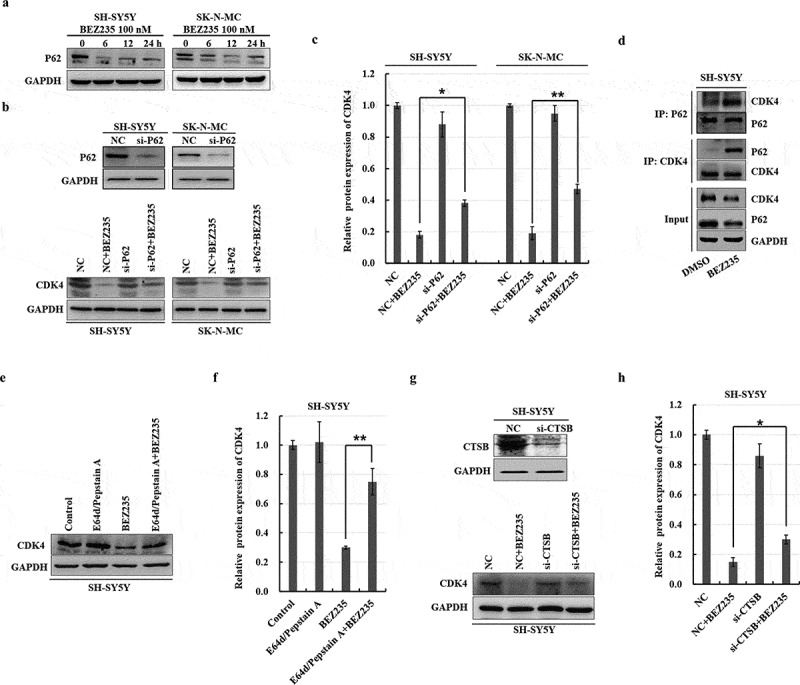
P62/CTSB mediated BEZ235-induced autophagic degradation of the CDK4 protein. (a) SH-SY5Y and SK-N-MC cells were incubated for the indicated times with 100 nM BEZ235 and then assessed by immunoblotting with an anti-p62 antibody. (b,c) NB cells were transfected with siRNA targeting p62 for 36 h and then treated with 100 nM BEZ235 for another 12 h. The cells were lysed and analyzed by immunoblotting with antibodies against CDK4. The results were normalized to NC group. (d) After NB cells were incubated for 12 h with 100 nM BEZ235, cell lysates were immunoprecipitated with an anti-p62 antibody or an anti-CDK4 antibody and then probed with an anti-CDK4 antibody or an anti-p62 antibody. (e,f) SH-SY5Y cells were pretreated for 1 h with pepstatin a (10 μM) and E64-d (10 μM) and subsequently treated with 100 nM BEZ235 for another 12 h. The cells were lysed and analyzed by immunoblotting with an anti-CDK4 antibody. The results were normalized to control group. (g,h) SH-SY5Y cells were transfected with siRNA targeting *CTSB* for 36 h and then treated with 100 nM BEZ235 for another 12 h. The cells were lysed and analyzed by immunoblotting with an antibody against CDK4. The results were normalized to NC group.

P62 (known as SQSTM1) is implicated in autophagic cargo recognition and is degraded in the final stages of autophagy during autolysosome degradation. A time-dependent reduction in p62 expression was demonstrated in both NB cell lines treated with BEZ235 (Figure 5a), consistent with the upregulation of LC3 II levels (Figure 3c,d). The CDK4 degradation induced by BEZ235 was reversed when p62 was silenced in NB cells by utilizing a siRNA approach (Figure 5b,c). Moreover, immunoprecipitation assays indicated that the interaction between p62 and CDK4 was enhanced after exposure to BEZ235 in SH-SY5Y cells (Figure 5d). These findings implied that BEZ235-induced CDK4 binds to the autophagy adaptor protein p62 in NB cells.

To validate the functional role of cathepsins in the degradation of CDK4 induced by BEZ235, lysosome-specific protease inhibitors E64d and pepstatin A were administered to SH-SY5Y cells. Importantly, suppression of cathepsins stabilized the CDK4 protein levels and reversed BEZ235-mediated CDK4 degradation (Figure 5e,f). Herein, we mainly focused on the regulatory effect of CTSB on CDK4 degradation. We observed attenuation of CTSB by siRNA resulted in a partial rescue of the BEZ235-induced CDK4 downregulation in SH-SY5Y cells (Figure 5g,h). Altogether, these results indicated that CDK4 enter lysosomes and undergo degradation in a CTSB-dependent manner.

### Autophagy-lysosome system mediates BEZ235-triggered CDK4 degradation in NB xenograft tumors

**Figure 6.**
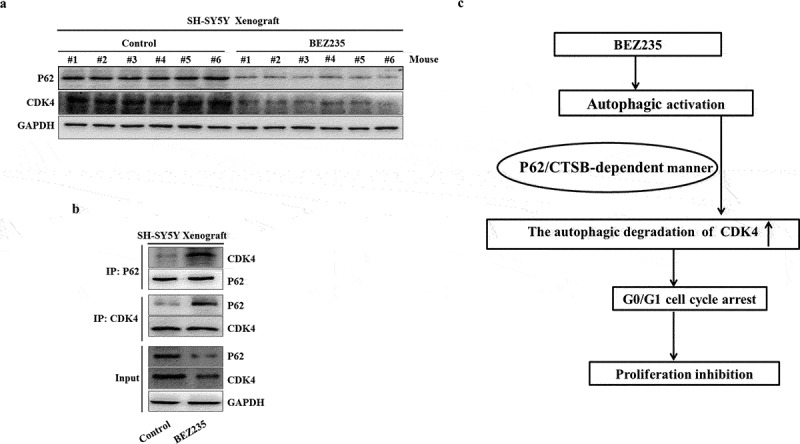
BEZ235-induced autophagic degradation of the CDK4 protein in NB xenograft tumors. (a) Tumor extracts were lysed and analyzed by immunoblotting with antibodies against p62 and CDK4 proteins. (b) Tumor extracts were immunoprecipitated with an anti-p62 antibody or an anti-CDK4 antibody and then probed with an anti-CDK4 antibody or an anti-p62 antibody. (c) Schematic presentation of the degradation of CDK4. BEZ235 induces the interaction of p62 with CDK4 and the subsequent autophagic degradation of CDK4 in a ctsb-dependent manner.

We have presented that antitumor activity of BEZ235 in mouse xenograft models of NB in our previous study about tumor size, weight, organ toxicity^19^(Fig. s5). To determine the association of autophagy with CDK4 protein degradation, an NB xenograft mouse model was generated. In agreement with the blockade of the G1 cell cycle in vitro, BEZ235 also reduced the expression of CDK4 in xenograft tumors, accompanied by a decrease in the autophagic protein marker p62 (Figure 6a). Furthermore, the enhanced interactions between p62 and CDK4 were similarly assessed by immunoprecipitation in the BEZ235-treated group (Figure 6b), which demonstrated that the autophagy-lysosome system mediates BEZ235-triggered CDK4 degradation. Together, these results revealed that BEZ235-induced autophagy plays a critical role in CDK4 degradation and tumor proliferation inhibition in NB xenografts.

## Discussion

CDK4 has been associated with poor prognosis and decreased survival in established tumors, including NB and its inhibition or degradation leads to G0/G1 phase arrest.^20,21^ The current strategies targeting CDK4 mainly focus on elimination of CDK4 catalytic activity via several potential CDK4 inhibitors. Disappointing results were observed for CDK4 inhibitors in terms of clinical toxicity and acquired resistance.^12,22^ Thus, the scheme of selective CDK4 degradation is appealing. Prior reports have demonstrated that the ubiquitin proteasomal system is responsible for CDK4 protein degradation in several tumors.^14,15,23^ In our work, we identified for the first time that BEZ235, as a potent dual PI3K and mTOR kinase inhibitor, induces CDK4 autophagic degradation in a P62/CTSB-dependent manner. Currently, the autophagy-lysosome pathway, another significant process in oncoprotein stability, has not been reported to play a role in CDK4 regulation in previous literatures.

CDK4 and members of the cyclin D family assemble into a complex with kinase activity to promote phosphorylation of Rb. The p-Rb results in the release of active nuclear transcription factor E2F which enters the nucleus, binds to a series of promoter regions, promotes downstream gene expression, and drives the progression of the G1-to-S cell cycle.^24^ The role of CDK4 via p-Rb in G1-to-S cell cycle progression is consistent with our study, in which we have shown that BEZ235 downregulates CDK4 protein, decreases p-Rb and thus induces G0/G1 cell cycle arrest in NB cells (Figure 1).

CDK4 protein is held at a constant level during cell cycle progression, which is regulated by periodical and coordinated protein synthesis and degradation.^25^ We have shown that BEZ235 induces autophagy and decreases CDK4 protein in NB, but it remains unclear about whether autophagy regulates CDK4 protein stability. Autophagic degradation of CDK4 is a novel mechanism, as there is evidence that ablating autophagy-related genes and autophagy-lysosomal inhibitors could obviously rescue CDK4 but the proteasome inhibitor could not recover the reduction of CDK4 in BEZ235-incubated NB cells. The other pathways could be involved in the reduction of CDK4, such as calpain and the midnolin proteasome pathway. This is suggested by the mild rather than total rescue of CDK4 levels observed with autophagy ablation. Surprisingly, the percentage of BEZ235-treated cells in the G0/G1 phase was partially decreased (Figure 4g) in SH-SY5Y cells after *Atg7* ablation. And a fully restored cell cycle profile is observed in SK-N-MC cells (Figure 4h) due to knockdown *Beclin-1*. The probable reasons for the differences are based on the different cell lines and autophagy-related gene. Further studies are needed to address the connection between the mild rescue of CDK4 levels and the fully restored cell cycle profile in SK-N-MC cells.

There is the crosstalk between CDK4 and the PI3K/mTOR pathway whose inhibition impacts CDK4 function via autophagy activation.^26,27^ Autophagy is a lysosomal proteolytic process that is crucial for degradation of damaged cellular components.^28^ Autophagic degradation of oncoproteins is a potential and novel approach for malignancies.^29,30^ Our subsequent experiments elaborate the particular modulatory mechanism of autophagy in BEZ235-induced CDK4 degradation. Obviously, p62 is required for constitutive autophagic degradation of CDK4, as it was illustrated that an enhanced interaction between p62 and the CDK4 proteins was observed and the CDK4 degradation induced by BEZ235 was restored when p62 was silenced in NB cells. Analogous results regarding the correlation of p62 and CDK4 were observed in an NB xenograft mouse model in vivo. P62/SQSTM1, as a cargo receptor protein, can bind directly to LC3 to promote the degradation of polyubiquitinated protein targets via macro-autophagy.^31^ As expected, the ubiquitylation level of CDK4 with the treatment of BEZ235 is enhanced (Fig. s4). Further research is necessary for associated ubiquitin ligases, types and sites.

Autophagy is an evolutionarily conserved pathway that delivers substrates to lysosomes for degradation.^32^ Moreover, intensive studies have shown that CTSB, as a notable lysosomal protease, is broadly upregulated in cancer and is essential for autophagic flux.^33–35^ Our data also implies CDK4 is a downstream substrate of CTSB in the autophagy process, as supported by our experiments in which selective knockdown of *CTSB* resulted in the partial reversal of CDK4 degradation. Additionally, LAMP2, a lysosomal marker, colocalizes with CDK4 in BEZ235-treated SY-SH5Y cells (Fig. s3). Based on these findings, we demonstrate that BEZ235 induces CDK4 binding to p62 to enter the autophagy-lysosome and then is degraded in a CTSB-dependent manner. Further investigations to decipher whether the interaction of p62 with CDK4 is direct and how BEZ235 initiates the interaction of p62 with CDK4 are necessary.

The roles of autophagy in cellular proliferation, differentiation, and cell death have been established in NB tumorigenesis and progression.^36,37^ Numerous studies have raised the potential of modulating the autophagic degradation of cell cycle-related protein, such as CDK2, cyclin D1, to promote drugs-induced antiproliferation effect.^38,39^ However, the roles and effects of autophagic degradation of CDK4 remain to be poorly understood in NB cells. In this report, we have affirmed that BEZ235-mediated CDK4 protein degradation, G0/G1 cell cycle arrest and proliferation inhibition which could be reversed by the autophagy ablation in NB cells. Our results emphasize the important role of autophagic degradation of CDK4 and it could be a potential avenue for NB treatment.

Of note, the SK-N-MC cell is now widely recognized as originating from an Askin’s tumor instead of NB. The mechanism of BEZ235-induced autophagic degradation of CDK4 has also been established in other tumor cell models, such as SK-N-MC, and NB cells like SH-SY5Y, which shows that the mechanism of autophagic degradation of CDK4 induced by BEZ235 is reliable and applicable. In addition, the doubling time of SH-SY5Y is within 24 h in our experimental condition (MEM/F12 + 15%FBS) which is different from previous reported long doubling time of SH-SY5Y cells.^40^ The reason for the difference may be the cell growth status and nutritional conditions.

## Conclusion

Taken together, our findings identify CDK4 as a critical target for autophagic degradation via a P62/CTSB-dependent mechanism, which is essential for the anti-proliferation effect of BEZ235 in NB cells (Figure 6c). These findings shed new light on the regulation and function of CDK4, providing a new clue for the rational utilization of the therapeutic drugs for modulation of autophagy in NB.

## Methods

### Cell lines, antibodies, and reagents

SH-SY5Y and SK-N-MC cell lines were purchased from the Shanghai Institutes for Biological Sciences (Shanghai, China) and were cultured in MEM/F12 medium (Hyclone, USA) containing 15% fetal bovine serum (Gemini, USA) at 37°C and 5% v/v CO_2_. The primary antibodies (all from Cell Signaling Technology) were as follows: CDK4, P62, Beclin1, LC3B, p-Rb (Ser608), CTSB, LAMP2. Anti-GAPDH, anti-rabbit HRP and anti-mouse HRP were purchased from AB clonal Biotechnology. BEZ235 was obtained from Novartis (Selleck, China) and dissolved in DMSO at a stock concentration of 1 mmol/L. Chloroquine (CQ), 3-MA, Pepstatin A, and E-64d were purchased from Sigma-Aldrich. Cycloheximide (CHX) was obtained from Med Chem Express. CQ and 3-MA were dissolved in sterile H2O to stock concentrations of 20 mmol/L and 100 mmol/L, respectively. CHX was dissolved in DMSO at a stock concentration of 100 mg/mL.

### Cell counting kit-8 assay

Cell viability was determined using the Cell Counting Kit-8 reagent (Dojindo, Japan). The NB cells (5 × 10^3^ cells/well) were seeded in 96-well plates in triplicate and treated with the indicated concentration. The transfected cells were treated with DMSO or 100 nmol/L BEZ235 for 12 h. The CCK-8 solution was added to each well and incubated for 4 h. The absorbance was obtained at 490 nm using a microplate reader (Bio-Rad, USA).

### Cell cycle analysis

The cells (1 × 10^6^) were harvested and were fixed in 75% ethanol overnight at 4°C after washing twice with cold PBS. Then, the cells were resuspended in propidium iodide/RNaseA-mixed staining solution (Beyotime, China) after washing twice with cold PBS, and incubated for 30 min at room temperature in the dark. The DNA content was detected by FACScan flow cytometer (BD Biosciences, USA). The results were analyzed by *ModFit 5.0* software (Verity Software House, USA)

### Western blotting

The total protein was extracted with RIPA lysis buffer (Beyotime, China) containing protease inhibitor and phosphatase inhibitor (Roche, Switzerland). The BCA Protein Assay Kits (ThermoFisher, USA) were used to perform standard curves to quantify the total protein before western blotting experiments. The 50 μg of protein from each group was separated by SDS-PAGE and transferred to PVDF membranes. Membranes were blocked in 5% nonfat milk at room temperature for 1 h and incubated with primary antibodies (1:1000) overnight at 4°C. The membranes were then washed with TBST and probed for 2 h at room temperature with secondary antibodies (1:3000). The bands were visualized using a chemiluminescent HRP Substrate (Millipore, USA) and captured with a digital imager (FUSION FX7, France). The optical density of the protein bands and the relative protein expression were quantified by Image J software (NIH, USA) and the results were normalized to the control group or GAPDH.

### Immunoprecipitation assays

The NB cells or tissues were lysed ice-cold RIPA lysis buffer containing protease and phosphatase inhibitors on ice for 30 min. The lysates were clarified by centrifugation at 10,000 ×g for 15 min at 4°C. The 50 μl lysate was used for western blot analysis and the remaining lysate (400 μl, 500 µg) was used for immunoprecipitation assays. The lysates were precleared with 20 µL 50% protein

A/G agarose slurry for 2 h with rotation at 4°C. Then, the lysate supernatants were transferred to another tube and incubated overnight at 4°C with 2 μg anti-p62 antibody, or anti-CDK4 and 30 µL protein A/G agarose. After washing four times with ice-cold lysis buffer by centrifugation at 5,000 rpm for 5 min, the samples were eluted in 2 × SDS loading buffer by boiling for 5 min and subjected to immunoblot analysis with anti-p62, anti-CDK4 or anti-ubiquitin antibodies.

### RNA extraction and RT-PCR

Total RNA in NB cell lines was extracted using RNAiso Plus (TaKaRa, Japan). The PrimeScript RT Master Mix (TaKaRa, Japan) was performed to synthesize cDNA. The quantitative PCR was amplified from cDNA using the specific primers and 2 × SYBR Premix ExTaq™ II (TaKaRa, Japan) in a Real-Time PCR Detection System (Roche, Switzerland) according to the manufacturer’s protocols. Related gene expression was calculated using 2^−ΔΔCt^ method after normalization to *β-actin*. The primers were designed and synthesized by Sangon Biotech Co., Ltd. (Shanghai, China). *CDK4* (forward, 5´-CTG GTG TTT GAG CAT GTA GAC C-3´; reverse 5´-GAT CCT TGA TCG TTT CGG CTG-3´). *β-actin* (forward,5´-ATA GCA CAG CCT GGA TAG CAA CGT AC-3´; reverse 5´-CAC CTT CTA CAA TGA GCT GCG TGTG-3´.

### Lentiviral transduction

The shRNA pGIPZ lentiviral constructs were obtained from Hanbio (China). The sequences used are as follows: a. *Beclin-1*#1 (5’-CCC GTG GAA TGG AAT GAG ATT-3’), *Beclin-1*#2 (5’-GCT TGG GTG TCC TCA CAA TTT-3’); b. Scrambled (5’-GTG GAC TCT TGA AAG TAC TAT-3’). c. *Atg7*#1 (5’-ACC ACC AGT TCA GAG CTA A-3’), *Atg7*#2 (5’-ACC AGT TCA GAG CTA AAT A-3’). The pGIPZ vectors and packaging plasmids (psPAX2, pMD2.G) (Life Technologies, USA) were co-transfected into packaging 293T cells using Lipofectamine-3000 (Life Technologies, USA). After 48 h, the viral supernatants were collected and used to infect SH-SY5Y cells. Stable clones expressing the shRNAs were obtained via 5 µg/ml puromycin dihydrochloride (Santa, USA) selection. SH-SY5Y cells transfected with the pcDNA3.1-vector and the pcDNA3.1-CDK4 plasmids (Sangon Biotech, China) using Lipofectamine-3000 were cultured for 48 h. The successfully transfected survival cells were obtained via 500 µg/ml G418 (Solarbio, China) for 24 h. The exclusive transfected cells were subjected to treatment with BEZ235 and the next cell cycle analysis. The *CDK4* sequences: 5’-CTT GGT ACC GAG CTC GGA TCC GCC ACC ATG GCT ACC TCT CGA TAT-3’.

### RNA interference

Small interfering RNAs (siRNAs) targeting human *CTSB*, *p62*, and negative control siRNA were synthesized by Sangon Biotech (China). The sequences used are as follows: *CTSB* (5’-UGG UCA ACU AUG UCA ACA ATT-3’); *p62* (5’-GAA UCU ACA UUA AAG AGA ATT-3’); negative control (5’-UUC UCC GAA CGU GUC ACG UTT-3’). The NB cells were transfected with siRNA using Lipofectamine-3000 (Life Technologies, USA) according to the manufacturer’s instructions.

### Immunofluorescence assays

NB cells were cultivated in a 96-well plate. The cells were treated by DMSO or 100 nM BEZ235 for 12 h and were fixed with 4% para-formaldehyde/PBS for 15 min at room temperature after washing twice with PBS. The cells were then washed three times with cold PBS, permeabilized with methyl alcohol for 10 min and finally washed with 0.05% Tween-20/PBS for 5 min. The cells were blocked by 5% BSA/PBS (Sangon Biotech) for 2 h at room temperature then incubated with primary antibodies (LAMP2 and CDK4, 1:100) overnight at 4°C. After washing three times with 0.05% Tween/PBS, the cells were incubated with a FITC-conjugated secondary antibody (1:100) for 2 h at room temperature in a dark humid chamber. The cell nuclei were stained with DAPI (Sigma-Aldrich, D9542). The cells were visualized by a fluorescence microscope (Nikon Eclipse Ti, Japan).

### Xenograft model of NB cancer

Animal experiments were approved by the Institutional Animal Care and Use Committee (IACUC) of Shanghai Jiao Tong University. Five- to six-week-old female BALB/c nude mice were obtained from Shanghai Slac Laboratory Animal Company. Mice were housed under standard conditions and provided with food and water ad libitum. A total of 5 × 10^6^ SH-SY5Y cells suspended in 100 μl of sterile PBS were inoculated subcutaneously into the right flanks of nude mice. When tumors reached an average volume of 50 mm^3^, the mice were randomized into two groups (*n* = 8 for each group): control (DMSO) and BEZ235 (20 mg/kg/d, i.p.). Tumor volumes were measured using caliper measurements once every day and calculated with the formula: V = a^2^×b × 0.4 where a is the smallest diameter and b is the diameter perpendicular to a. The body weight, feeding behavior and motor activity of each animal were monitored as indicators of general health. After 3 weeks, mice were euthanized. Tumor xenografts were excised, weighed and homogenates were prepared for western blotting.

### Statistical analysis

All experiments were analyzed in three independent replicates and the error bars represent the standard deviations. Statistical analyses were performed using Microsoft Office Excel 2013 (Microsoft, USA) and GraphPad Prism 7.0 software (GraphPad Software, USA). The unpaired t-test was used to compare the differences between two groups. *p* < .05 was considered statistically significant. *p < .05, **p < .01.

## List of abbreviations

**Table:** 

NB	neuroblastoma
CDK4	cyclin dependent kinase 4
E2F	the E2 factor
CTSB	cathepsin B
LC3	microtubule-associated protein light chain 3
SQSTM1	sequestosome 1
siRNA	small interfering RNA
shRNA	short hairpin RNA
PI3K	phosphatidyl inositide 3-kinase
mTOR	mammalian target of rapamycin
RT-PCR	reverse transcription-polymerase chain reaction
BCA	Bicinchoninic Acid

## Supplementary Material

Supplementary Figure Captions2 clean.docx

FIGs4.tiff

FIGs3.tiff

FIGs1.tiff

FIGs2.tiff

FIGs5.tiff

## Data Availability

The datasets generated and analyzed during the current study are available from the corresponding author on reasonable request.
