# Supplementary material for: Autophagic degradation of CDK4 is responsible for G0/G1 cell cycle arrest in NVP-BEZ235-treated neuroblastoma
Source: Cancer Biol Ther. 2024 Aug 1;25(1):2385517. doi: 10.1080/15384047.2024.2385517 (PMC11296530; doi:10.1080/15384047.2024.2385517)
Supplement: Supplementary Figure Captions2 clean.docx [file KCBT_A_2385517_SM5480.docx]

**Supplementary Figure Captions**

**Fig. s1. The autophagy inhibitors could reverse G0/G1 phase arrest mediated by BEZ235.**

SH-SY5Y cells were pretreated for 1 h with an autophagy inhibitor (20 μM CQ or 2 mM 3MA) and subsequently treated for another 12 h with 100 nM BEZ235. Cell cycle distribution was shown by flow cytometric analysis.

**Fig. s2. The cell cycle distribution of NB cells treated with or without NVP-BEZ235 after knockdown of autophagy-related genes.**

SH-SY5Y and SK-N-MC cells were transfected with shRNA specifically targeting *Atg7* or *Beclin-1* and transfected cells with individual shRNAs were subsequently treated for 12 h with DMSO or 100 nM BEZ235. Cell cycle distribution was determined by flow cytometric analysis in the Fig. 4g and h.

**Fig. s3. The colocalization of lysosomal marker LAMP2 and CDK4**.

SH-SY5Y cells treated with DMSO or 100 nM BEZ235 for 12 h were stained with anti-CDK4 (red) and anti-LAMP2 (green) and signals were detected by fluorescence microscopy. Merged panels indicate overlapping images of the two fluorescing signals and arrows show the colocalization of CDK4 and lysosomes. Scale bars: 20 μm.

**Fig. s4. The ubiquitin levels of SH-SY5Y cells with or without BEZ235.**

After SH-SY5Y cells were incubated for 12 h with DMSO or 100 nM BEZ235, cell lysates were immunoprecipitated with an anti-CDK4 antibody and then probed with anti-ubiquitin antibodies.

**Fig. s5. The weight and volume of tumors in NB xenograft tumors.**

The weight and volume of SH-SY5Y xenograft tumors were measured in DMSO and BEZ235 treated groups.
