# Supplementary figures and images for: Autophagic degradation of CDK4 is responsible for G0/G1 cell cycle arrest in NVP-BEZ235-treated neuroblastoma
Source: Cancer Biol Ther. 2024 Aug 1;25(1):2385517. doi: 10.1080/15384047.2024.2385517 (PMC11296530; doi:10.1080/15384047.2024.2385517)

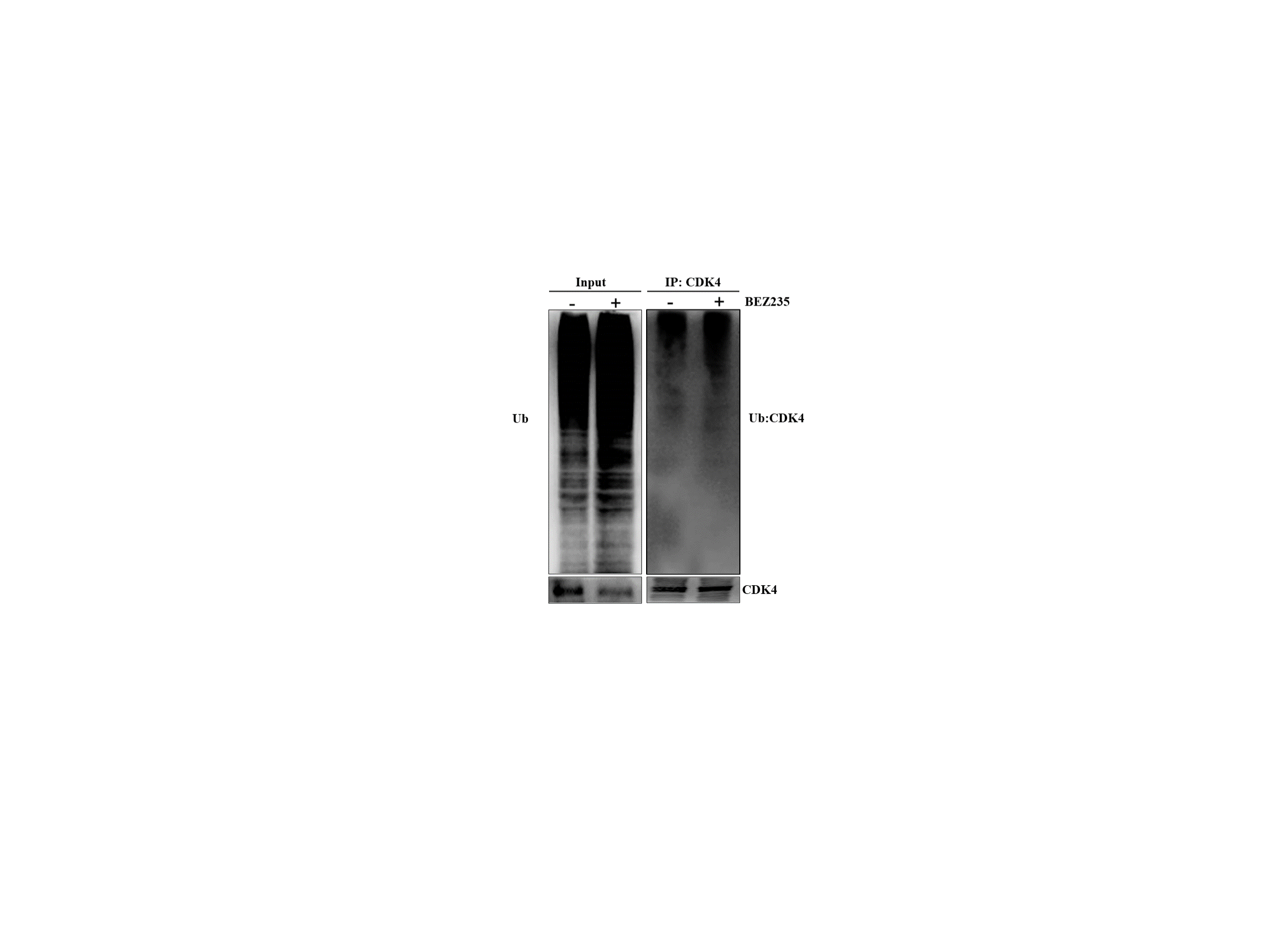

Supplement: FIGs4.tiff [file KCBT_A_2385517_SM5479.tiff]

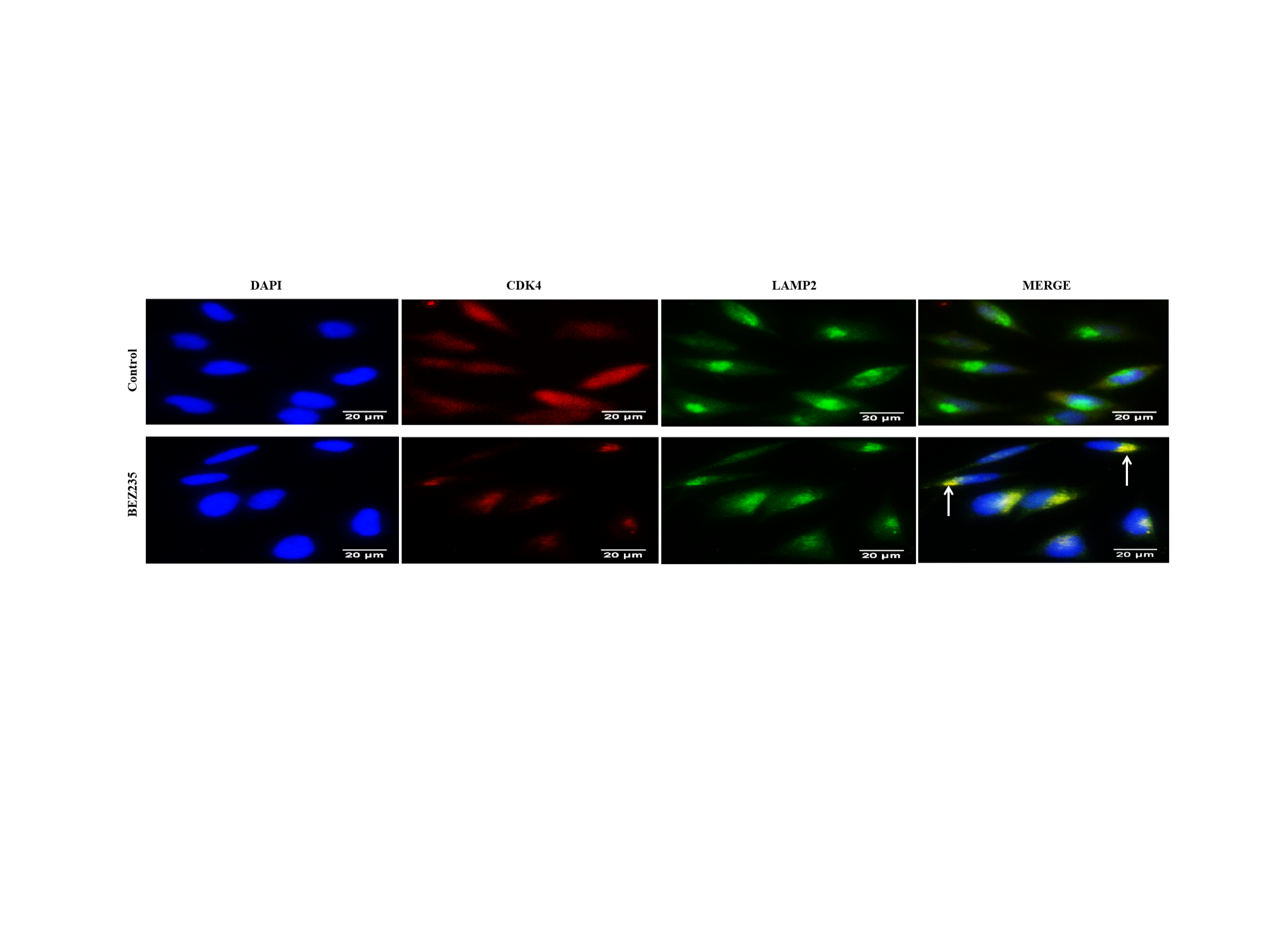

Supplement: FIGs3.tiff [file KCBT_A_2385517_SM5478.tiff]

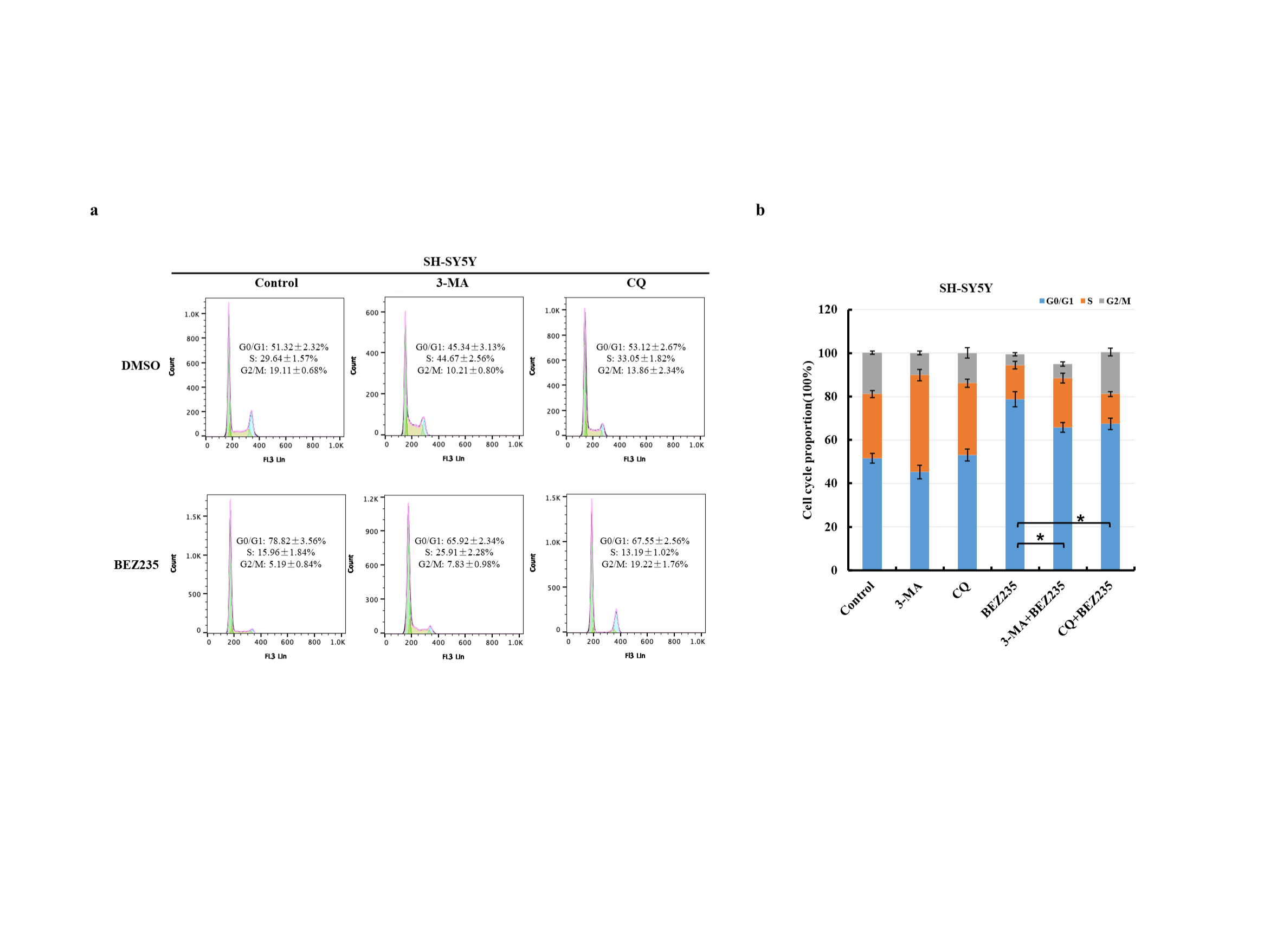

Supplement: FIGs1.tiff [file KCBT_A_2385517_SM5477.tiff]

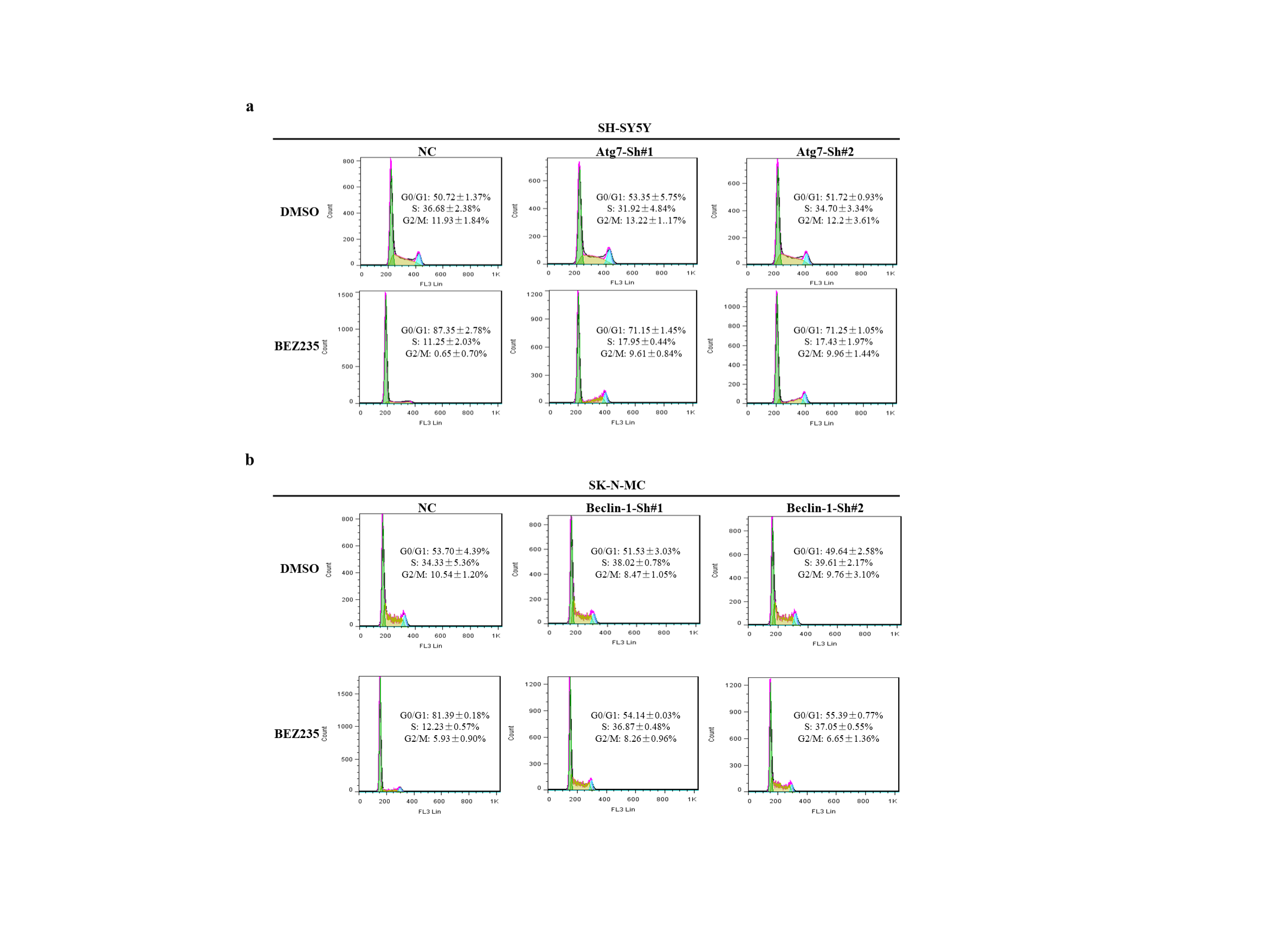

Supplement: FIGs2.tiff [file KCBT_A_2385517_SM5476.tiff]

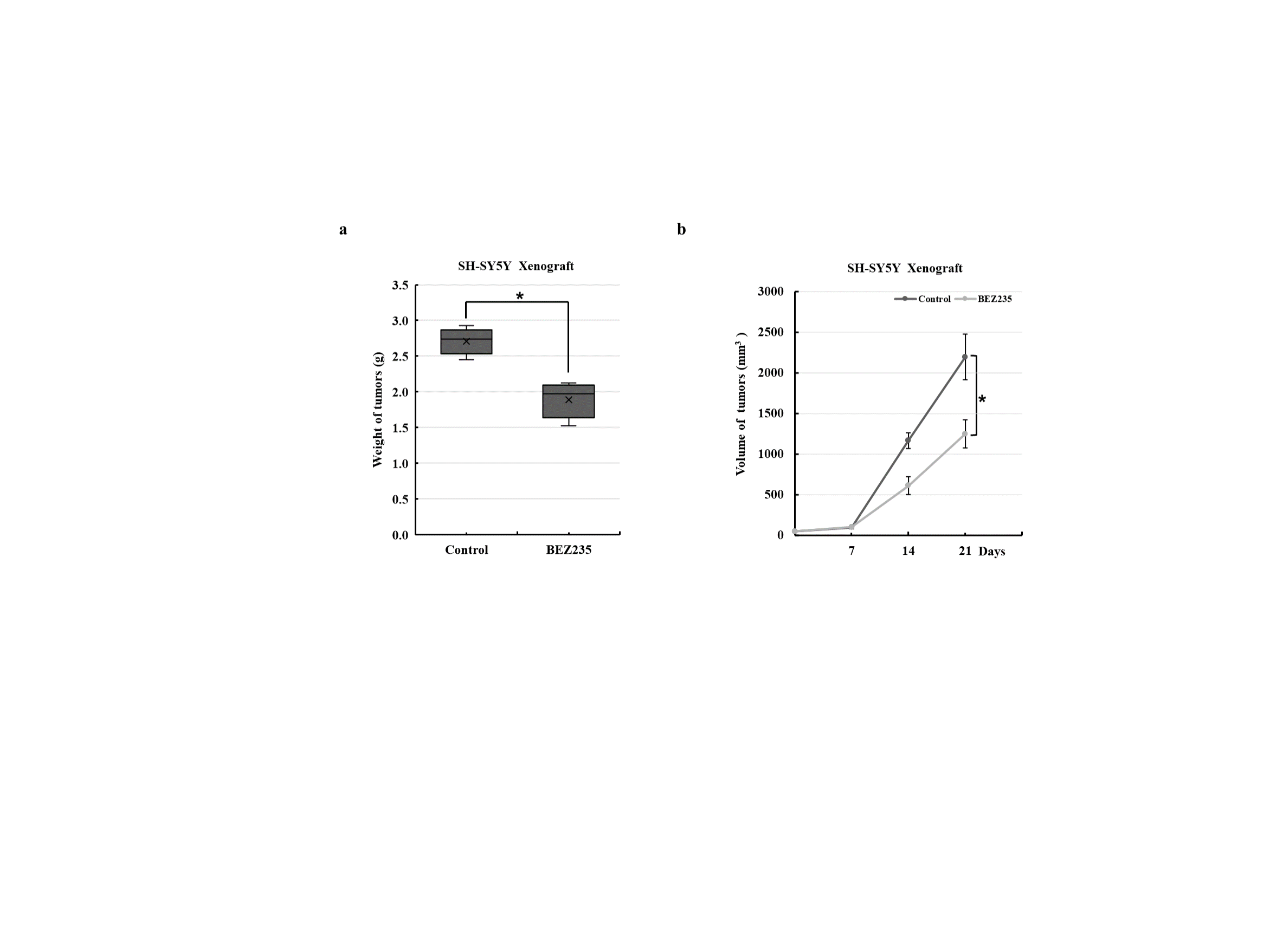

Supplement: FIGs5.tiff [file KCBT_A_2385517_SM5475.tiff]
